# Efficacy of Electroconvulsive Therapy for the Treatment of Movement Disorders: A Literature Review

**DOI:** 10.7759/cureus.36634

**Published:** 2023-03-24

**Authors:** Nazar Muhammad, Nathaniel Brooks III, Lauren Chatham, Anthony Chatham, Purushothaman Muthukanagaraj

**Affiliations:** 1 Psychiatry, Cornerstone Family Healthcare, New York, USA; 2 Private Privatice Psychiatry, Out Patient Psychiatrist, Phoenix, USA; 3 Forensic Psychiatry, Georgia Regional Hospital Atlanta, Decatur, USA; 4 Psychiatry and Behavioral Science, Emory University, Atlanta, USA; 5 Psychiatry, State University of New York Upstate University Clinical Campus Binghamton, Binghamton, USA; 6 Psychiatry, United Health Services, Binghamton, USA

**Keywords:** movement disorders, review article, treatment choices, psychiatric comorbidities, electroconvulsive therapy (ect)

## Abstract

Electroconvulsive therapy (ECT) is a safe and effective treatment modality for various psychiatric disorders. However, evidence suggests a putative role of ECT in treating movement disorders that are refractory to less invasive modalities. ECT is primarily used in treatment-resistant psychiatric disorders. However, growing evidence exists for its use in movement disorders with and without psychiatric comorbidity.

The primary objective of this systematic review was to examine the efficacy of ECT as a primary treatment modality for movement disorders. Relevant, peer-reviewed publications were retrieved from PubMed, SCOPUS, CINAHL, and PsycINFO. Keywords related to ECT and movement disorders were used as search phrases to identify relevant articles. A total of 90 articles that met the inclusion criteria were included in this review. Core findings were subsequently appraised on the role of ECT in treating movement disorders.

Inclusion and exclusion criteria were developed to guide the search and selection process. Sources that met the inclusion criteria were those published between 2001 and January 2023. Additionally, peer-reviewed journals published in the English language covering the role of ECT in movement disorders were deemed appropriate for inclusion. Sources published before 2001, written in a non-English language, and not from peer-reviewed journals were excluded from this systematic review. The exclusion criteria also entailed eliminating duplicates from the review list.

Most reviewed sources revealed that ECT improved outcomes in symptoms associated with various movement symptoms. However, ECT does not have a long-lasting impact on neuroacanthocytosis symptoms. Additionally, ECT is negatively associated with aggression and agitation, two of the most critical movement symptoms of Alzheimer’s disease.

Evidence affirms the efficacy of ECT in providing symptomatic relief for movement disorders aside from psychiatric comorbidities. This positive association highlights the need for randomized controlled studies to identify movement disorder sub-populations that may respond to ECT.

## Introduction and background

The effectiveness of electroconvulsive therapy (ECT) has been documented since the mid-twentieth century. Many regard ECT as a treatment option that should be used earlier in the treatment course of some forms of psychiatric illness and may provide more cost-effective care when used in this manner [1]. In the most general view, ECT can be defined as a type of medical treatment that is primarily utilized for treating severe psychiatric disorders and involves the administration of an electrical current to the brain while keeping the patient under general anesthesia [1]. The seizure activity associated with ECT is believed to change brain chemistry, thus improving the symptoms of psychiatric disorders [1,2].

ECT has been used for various disorders, including, but not limited to, catatonia, psychosis, depression, neuroleptic malignant syndrome, and status epilepticus. Although the exact mechanism of action leading to improvement is yet to be elucidated, multiple hypotheses exist. Numerous reports have been published about movement disorders with and without psychiatric comorbidity. This topic has attracted the attention of multiple researchers who have published findings to support or disapprove of the efficacy of ECT on movement disorders, such as Parkinson’s disease (PD) and Parkinsonian disorders, Huntington’s disease, and dystonia. Parkinsonian disorders involve neurological conditions that are characterized by the degeneration of nerve cells that are responsible for the production of dopamine, Huntington’s disease is a generic neurological caused by a mutation in the *huntingtin* gene leading to the abnormal production of protein, and dystonia is a neurological movement disorder that manifests itself in involuntary muscle contractions resulting in abnormal and twisting movements or postures. Despite this growing attention, systematic literature reviews appraising the quality of the extant body of evidence on the usefulness of ECT in managing these disorders as an alternative treatment intervention are lacking. To date, only two systematic reviews have assessed the available data on ECT and its role in the treatment of multiple movement disorders [2,3]. Hence, this systematic review fills the existing knowledge gap by exploring and evaluating the most recent literature on the efficacy of ECT in movement disorders with and without psychiatric comorbidity, secondary outcomes, and trends for treatment parameters.

## Review

Methods

Search Strategy

Four databases were used in identifying publications that could yield relevant data on the role of ECT in improving movement disorders: PubMed, SCOPUS, CINAHL, and PsycINFO. These databases were selected due to their richness in biomedical, healthcare, and medical research. The following phrases were used as keywords to obtain the most recent literature: “ECT and movement disorders,” “ECT and Parkinsonism,” “ECT and dyskinesia,” and “ECT and dystonia.” The identified keywords were copy-pasted directly to the search bar of each of the four databases repeatedly to amplify the chances of retrieving relevant publications. Each key phrase was searched separately to alleviate confusion and augment consistency. Articles from January 2001 to January 2023 were deemed appropriate and contemporaneous for inclusion in this review. Limiters were applied across the four databases to access the most suitable publications. The search process was also restricted to peer-reviewed English-language journals.

Inclusion and exclusion criteria were developed to guide the search and selection process. Sources that met the inclusion criteria were those published between January 2001 and January 2023. Additionally, peer-reviewed journals published in the English language and covering the role of ECT in movement disorders were deemed appropriate for inclusion. Sources published before 2001, written in a non-English language, and not from peer-reviewed journals were excluded from this systematic review. The exclusion criteria also entailed eliminating duplicates from the review list.

Search Outcomes

**Figure 1:**
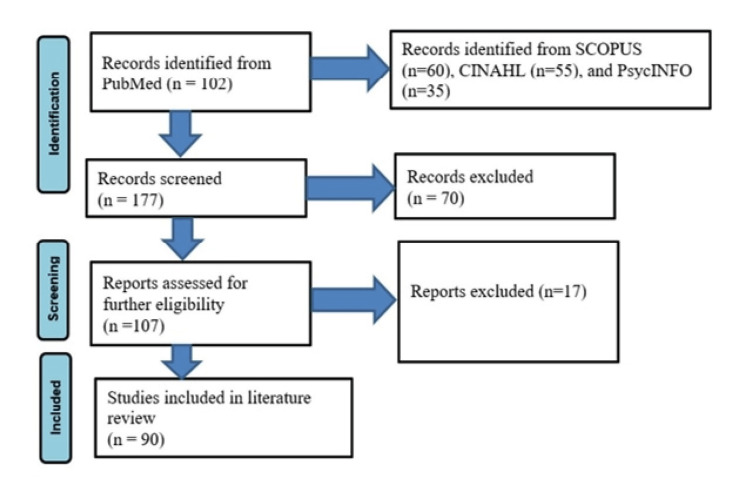
PRISMA flowchart: a summary of the search process. PRISMA: Preferred Reporting Items for Systematic Reviews and Meta-Analyses

The first search parameters yielded a total of 252 potential citations: PubMed (n = 102), SCOPUS (n = 60), CINAHL (n = 55), and PsycINFO (n = 35). Duplicates, non-English-language articles, pending publications, and abstracts from meeting proceedings were excluded (n = 75). Two reviewers (NB and AC) independently reviewed the titles, abstracts, and full texts of the remaining sources (n = 177) for relevancy. Subsequently, 70 sources that failed to meet the inclusion threshold were eliminated from the list. The remaining articles (n = 107) were analyzed further using their bibliographies to ascertain their suitability. Subsequently, 17 publications were eliminated because they were deemed not relevant enough. The remaining articles (n = 90) and their bibliographies were examined for case reports or case series on the use of ECT in patients with various movement disorders irrespective of the co-existence of a mood disorder. All sources were established to be suitable for data extraction. Thus, 90 studies were used for data extraction (Figure 1). The two reviewers collected and summarized the data in a table form. The third reviewer (LC) assessed tables for inconsistencies, which were clarified in the final list.

Results

A total of 90 articles that met the inclusion criteria were analyzed further to understand the extent to which they supported the relevancy of ECT in treating movement disorders. A systematic review of the articles uncovered the following themes: the potential of ECT to treat PD, the role of ECT in treating Tourette’s and other tic-like disorders, the role of ECT in treating tardive dyskinesia, and the role of ECT in treating other movement disorders mentioned in this article. This section systematically reviews these themes based on the re-analyzed data.

Parkinson’s Disease and Parkinsonian Disorders

Most reviewed literature showed a positive correlation between ECT and PD. While conducting this review, there were 32 publications with patients being treated with ECT and having PD or Parkinsonian disorder [4-36]. Two studies confirmed a positive relationship between ECT and PD, with significant improvements in the associated symptoms being reported [4,5]. One of them involved the use of ECT within two-week intervals in a patient experiencing primary parkinsonian syndrome (stage 3, onset at 39 years) and schizophrenia [4]. The patient’s Unified Parkinson’s Disease Rating Scale (UPDRS) score decreased from 80/132 to 40/132 as a result of the intervention [4]. The other one, at the same time, showed the effectiveness of ECT in treating psychosis and depression symptoms in 12 patients with the help of ECT sessions (12 ± 2.8) [5]. In particular, the patients enjoyed an improvement in the Brief Psychiatric Rating Scale (BPRS) and Hamilton Depression Rating Scale (HDRS) by 52% and 50% points, respectively [5]. Cases with documentation varied from weeks to years. The two retrospective studies included 32 patients [5,10]. There were comorbid depression and psychosis. In both studies, patients received bilateral frontotemporal treatment. The findings indicated a statistically significant reduction in Hoehn and Yahr (HY) scale and a decrease in levodopa daily equivalent dose within 5-30 weeks in followed-up patients [10].

In the 24 case reports, ECT managed to display impressive effectiveness in addressing comorbid diagnoses, including schizophrenia [4], post-traumatic stress disorder [6], bipolar disorder [7], drug-induced psychosis [8], bifrontal [9], and bilateral [10-26]. In most studies, there were improved psychiatric symptoms in all patients with the comorbid diagnosis. Several scales were used, including BPRS [5,10], HAM-D/HDRS [5,7], Clinical Global Impression [5], Montgomery-Asberg Depression Rating Scale [6], Global Assessment of Functioning Scale [10], Beck Depression Inventory [20], Sense Coherence [20], Quick Inventory Depressive Symptomatology [18], and Positive and Negative Syndrome Scale (PANSS) [25].

One study even collected data on improvement on the neuropsychiatric inventory scale, which evaluates behavioral symptoms associated with dementia [20]. The scientific papers examined in this review demonstrate that ECT is highly effective in addressing various movement disorder symptoms and other neuropsychiatric problems. However, there was no worsening of motor symptoms or monitoring for improvement in the two reports [14,22]. One case of Parkinson’s hyperpyrexia syndrome and treatment led to the resolution of neuroleptic malignant (NMS)-like symptoms [23]. In one case, the family decided to stop the application of bilateral ECT in a patient with PD, drug-induced psychosis, and NMS [24]. Three studies illustrated significant motor improvements in patients, such as stability over a five-year period with a low dose of levodopa (300 mg/day) [24], a decrease in Unified Rating Scale for Parkinsonism (URSP) and PANSS scores from 67 to 41 and from 82 to 40, respectively, owing to nine ECT sessions [25], and a reduction in the number of freezing episodes in the on phase following eight sessions of bitemporal ECT [27].

In three studies, there was documented motor improvement from no worsening, modest, to reductions in Barthel index, HY, and UPDRS [24,25,27]. In two studies, delusions, or depression with and without psychotic features, psychosis due to the medical condition, Capgras syndrome, catatonia, obsessive-compulsive disorder plus depression, unspecified psychosis, or a combination of anxiety were reported as comorbid diagnoses [27-29]. In two studies, improvements were observed clinically or reported as an increased level of functioning for several cases [22,27]. There was an improvement in all scales used; however, only measurements during the on phase showed statistical significance [27]. Lead placement varied between unilateral, left anterior, right temporal, and unspecified [28]. There was reported motor improvement in all cases, such as a reduction in the URSP score from 67 to 41 [27]. The length of motor improvement was not documented in some cases [29,30]. Motor improvement ranged from clinically observed, decreased UPDRS score, medication taper, and DBS turned off [29,30].

Five other publications established a positive association between ECT and improved motor manifestations in patients with PD as well as their depression and psychosis symptoms [31], impulse control disorders [32], psychiatric symptoms [33], refractory psychiatric symptoms [34], and neuroleptic malignant syndrome [35]. A study involving 15 PD patients revealed that besides psychiatric symptoms, the therapy led to significant improvements in motor functionality after the end of the sixth and tenth weeks [31]. However, the researchers underscored the need for patients to undergo traditional treatment with ECT to record long-lasting outcomes. One case study investigated the role of ECT in treating depression and neuroleptic malignant syndrome among PD patients [32]. The report focused on a 64-year-old woman with PD diagnosed with state 1V severity on the HY scale. The patient was placed under ECT to appraise its efficacy in managing the two symptoms. The patient’s depression and malignant syndrome, which is a life-threatening condition characterized by autonomic dysfunction, altered mental status, and muscle rigidity as a result of dopamine receptor blockade in the brain, improved significantly after receiving five sessions of modified ECT [32].

A similar outcome was reported in a systematic review aimed at assessing the efficacy of ECT in managing non-motor symptoms, such as depression and psychosis, among PD patients [33]. The review showed that PD patients treated with ECT reported improved motor capabilities, with those without psychiatric symptoms demonstrating better outcomes. Additionally, the study also established that ECT enhanced depression and psychosis symptoms, without worsening cognitive functioning. The positive effect of ECT on PD patients was established in another primary study investigating the therapy’s role in treating motor and non-motor symptoms [34]. The study utilized the HY scale for staging the disease and the UPDRS score for examining motor symptoms. The mean scores in the two measures decreased significantly after the ECT intervention. Notably, impulse control disorders vanished following the completion of the ECT sessions in five patients who experienced them before ECT; moreover, PD patients reported improvements in psychiatric and motor symptoms that lasted for more than a year in nine and five cases, respectively [34]. Improved outcomes in motor and psychiatric symptoms lasted for a year while the daily dose of anti-PD drugs was reduced substantially.

Another study analyzing clinical data for PD patients confirmed a positive association between ECT and psychiatric and motor symptoms [35]. The researchers analyzed clinical data from a Vancouver-based university for PD patients receiving ECT between 2014 and 2018. The majority of the patients reported improved outcomes in depressive and motor symptoms after undergoing ECT. Similarly, a study involving eight patients with dementia with Lewy bodies demonstrated a positive impact of ECT sessions conducted between 2013 and 2019 on psychotic symptoms as per the BPRS scale (p < 0.018) [36]. All patients showed improvements in psychotic symptoms after being administered ECT which lasted for more than one year after the end of the intervention.

Drug-Induced Parkinsonism

During the review, there were three published reports regarding patients with drug-induced Parkinsonism and ECT treatment, with all showing a decrease in their symptoms [37-39]. One of the studies reported a complete resolution of dementia after 19 ECT treatments in a PD female with a major depressive episode and psychotic features [37], while another study reported an improvement in drug-induced parkinsonism following eight bilateral ECT sessions, which lasted for six months after the end of the treatment [39]. The patients received 19, 10, and eight treatments of ECT, respectively [37-39]. One patient’s movement disorder improved such that he had only mild bradykinesia at the time of discharge [37]. Mood symptoms also resolved following treatment [31]. Another case described an intellectually disabled patient who was started on an atypical antipsychotic due to aggressive behaviors, and treatment led to Parkinsonian symptoms. The medication was discontinued, and ECT was initiated. Following three of the ten treatments, the same antipsychotic was restarted. There was no recurrence of Parkinsonian symptoms or maintenance ECT in six months of follow-up [38]. An elderly PD patient with long-standing paranoid schizophrenia and drug-induced parkinsonism received eight modified bilateral treatments over three weeks without any maintenance treatment [39]. After ECT, the patient was determined to have entered clinical remission with only minimal Parkinsonian symptoms, and he did not have a recurrence over the six-month follow-up period.

Tardive Dyskinesia

During the review, there were five case reports regarding patients with tardive dyskinesia and ECT, with all showing improvement [40-44]. In particular, such improvements involved the sustention of blepharospasm remission until three months after the maintenance of ECT [40], improvements in psychotic and dyskinetic movements in a female patient with tardive dyskinesia for eight years [41], improvements in tardive dyskinesia in a PD patient with depression [42], a decrease in the Abnormal Involuntary Movement Scale (AIMS) score from 19.1 ± 4.7 to 9.6 ± 4.2 in seven out of 18 patients [43], and improvements in drug-induced blepharospasm with ocular dystonia owing to ECT [44]. AIMS improvement was concurrent with the improvement in the patient’s psychotic and mood symptoms [40-42]. Patients received bilateral [40,41,43], right unilateral [44], and bifrontal [42] lead placement. There was one retrospective study including 18 patients receiving ECT for tardive dyskinesia. Their psychiatric comorbidity included schizophrenia or unipolar depression. One of these studies reported a significant improvement in global functioning scores [43]. Two cases reported remission followed by recurrence of symptoms after m-ECT was discontinued [40,44]. Three case reports showed a decrease of at least 8 points in the AIMS score [45], with two showing a decrease of 10 or more points [42,46].

Two other studies analyzed in this systematic review affirmed a positive implication of ECT on tardive dyskinesia [45,46]. A case report examining the efficacy of ECT on a 74-year-old female patient with tardive tremors and major depressive disorder confirmed this impact [45]. The patient, who underwent 10 sessions of ECT to treat major depressive symptoms, reported significant improvement in tardive tremor symptoms. This outcome validates the role of ECT in managing tardive tremors among patients with major depressive disorder. Similarly, a systematic review analyzing 23 primary studies established ECT as an effective intervention for major depressive disorder accompanying tardive tremors [46]. The findings affirmed ECT as an alternative treatment for major depressive disorder in patients with tardive tremors. However, this intervention was found to be more effective when patients reported refractory mood or affective disorder. Nevertheless, the researchers underscored the need for further research to establish the therapy’s efficacy, safety, and tolerability.

Tourette’s and Other Tic-Like Disorders

There has been limited data on the effects of ECT on patients with Tourette’s syndrome or other tic-like disorders. During this review, there were eight case reports of patients with tic-like disorders, including Tourette’s, being treated with ECT [47-54]. There was a positive effect of ECT in all cases regardless of lead placement, such as a two-year remission after seven ECT sessions [49], the elimination of any symptoms of Gilles de la Tourette’s syndrome for five months after ECT [52], and the resolution of symptomology in a patient who suffered both from obsessive-compulsive disorder and Tourette’s syndrome [54].

Cases showed a reduction in the severity of symptoms to complete remission. All cases with documented psychiatric comorbidity reported symptom improvement after treatment with ECT [47-54]. Three patients with subtypes of multisystem atrophy improved both motor and psychiatric symptoms following ECT [48,50,53], such as a two-year remission in Gilles de la Tourette syndrome after seven ECT sessions [53]. Two reports involved subclasses of dystonia (dopa-responsive dystonia and primary cervical dystonia), delusional depression, and agitation [54,55]. There were no adverse effects on motor symptoms in the case of dopa-responsive dystonia. However, before ECT treatment, symptoms were controlled pharmacologically [54]. The case of cervical dystonia also had bilateral pallidal deep brain stimulators placed, contributing to stable movement symptoms. Both cases of dystonia showed improvement in psychiatric symptoms [54,55].

A study examining the most effective therapy to augment clozapine in treating tardive dyskinesia among patients with treatment-resistant schizophrenia established ECT as a viable option [55]. ECT was found to be the most effective therapy in supporting clozapine to treat tardive dyskinesia among individuals with drug-resistant schizophrenia. The study provides additional evidence on the role of ECT in augmenting pharmacological medication to treat drug-resistant schizophrenia. This relationship was in line with the outcome of a systematic review of previous medical reports [56], in which 70% of the reviewed reports indicated the effectiveness of ECT in reducing the symptoms of Tourette’s syndrome and related conditions.

Other Movement Disorders

The reviewed literature established 11 reports of patients with diagnoses including multisystem atrophy, Huntington’s disease, paroxysmal non-kinesigenic dyskinesia, essential tremor, and cervical dystonia [57-69]. After receiving treatment with ECT, patients experienced an improvement in psychiatric symptoms. Another case report involving a patient with neurological symptoms due to dopa-responsive dystonia reported a positive outcome in a 38-year-old woman with a history of dopa-responsive dystonia and chronic depression in terms of dyskinesia and dystonia (GDSTS, 2; UDRS, 1.4/44; fewer mood swings) [57]. The patient reported improved neurological outcomes after undergoing ECT, thus authenticating the intervention’s role in treating neurological symptoms among patients with dopa-responsive dystonia. Two cases involving a patient with Huntington’s disease experienced an improvement in movement disorder and psychiatric symptoms [58,59]. In the case discussing a patient with Paroxysmal non-kinesigenic dyskinesia and major depressive disorder, there was only significant improvement in psychiatric symptoms but not in dyskinesia [60]. Several cases showed different outcomes in neuroacanthocytosis symptoms among patients receiving ECT, although their results were inconclusive [61-69]. For instance, one of the studies reported the inability of ECT sessions to stop the progression of neuroacanthocytosis in a 24-year-old man with obsessive-compulsive disorder, moderate depressive disorder, and personality disorder [61].

The association between ECT and symptoms of mental health disorders was confirmed in a report focusing on depression, chorea, and psychosis in a patient with Huntington’s disease [70]. Two studies confirmed a positive relationship between ECT and psychiatric symptoms among patients with Huntington’s disease [70,71] while two others reported a negative relationship [72,73]. The patient reported significant improvements in severe depression, psychosis, and chorea after receiving ECT. The role of ECT was further authenticated by a retrospective case series [71]. Patients who received ECT reported better outcomes in depression, suicidal intentions, and agitation, resulting in successful hospital discharge. Consistently, a scoping review revealed that ECT reduces psychiatric symptoms associated with Huntington’s disease [74]. The results indicated that ECT improved patients’ depression when combined with venlafaxine. Thus, this study authenticated the positive association between ECT and improved Huntington’s disease symptoms. Despite these promising outcomes, a recent review of studies focusing on movement performance showed a negative association between ECT and psychiatric symptoms [72]. Notably, ECT was found to worsen movement functionality among patients with Huntington’s disease. Similarly, a patient report confirmed that ECT deteriorates movement abilities among patients with Huntington’s disease [73].

The efficacy of ECT in treating major depressive symptoms was validated by four additional studies [75-78]. One systematic study examined the efficacy of ECT in managing drug-resistant depressive symptoms [75]. The findings revealed that ECT improved drug-resistant major depressive symptoms among patients. A clinical study examining the efficacy of ECT in achieving long-term outcomes among individuals experiencing depressive symptoms and functional disability further supports these findings [76]. In this study, adults with major depressive episodes received ECT at an ambulatory clinic between September 2010 and November 2020. ECT was found effective in minimizing depressive symptoms and improving functional disability. Another clinical study established a significant impact of ECT on the white matter changes over time among major depressive disorder patients [77]. In this study, 29 patients with severe depression received ECT to augment inpatient treatment. The therapy transformed the integrity of the white matter over time, thus diminishing the possibilities of increased seizure activities. One controlled study reported that ECT improved suicidal ideation among major depressive patients [78]. ECT resulted in the improvement of psychiatric symptoms but continued to show progressive deterioration until death by the disorder.

Nevertheless, one case report failed to establish a statistically significant outcome on the effect of ECT on symptoms related to Alzheimer’s disease [79]. According to the findings, the intervention did not yield positive outcomes insofar as agitation and aggression are concerned.

Table 1 presents a synopsis of the reviewed studies.

**Table 1:** A synopsis of the reviewed studies. PD: Parkinson’s disease; ECT: electroconvulsive therapy; AD: Alzheimer’s disease

Authors and Year	Objective	Type of article	Results
Afshari et al. (2022) [31]	To examine the efficacy of electroconvulsive therapy in PD patients	Clinical trial	Significant improvements in motor functionality
Fukatsu and Kanemoto (2022) [36]	To evaluate the efficacy of ECT in improving psychotic clinical signs of dementia with Lewy bodies	Clinical trial	Positive psychotic symptoms after being administered ECT
McManus et al. (2022) [79]	To examine the efficacy of ECT in treating aggression and agitation among patients with AD	Clinical trial	The study did not show statistically significant outcomes
Yahya and Khawaja (2021) [46]	To analyze the use of ECT in the treatment of tardive dyskinesia	Case study analysis	Improvements in her tardive tremor symptoms
Yeh et al. (2020) [45]	This case report examines the efficacy of ECT on a 74-year-old female patient with tardive tremors due to major depressive disorder	Case report	Improvements in her tardive tremor symptoms
Roerig (2019) [55]	To examine the most effective therapy to augment clozapine in the treatment of tardive dyskinesia among patients with treatment-resistant schizophrenia	Exhaustive literature review	Improvements in her tardive tremor symptoms
Dos Santos-Ribeiro et al. (2018) [56]	To examine the proficiency of ECT on compulsive-related disorders, including Tourette’s	A systematic literature review	A positive outcome in Tourette’s syndrome and related conditions
Mowafi and Millard (2021) [70]	To examine the role of ECT in treating severe depression, chorea, and psychosis in a patient with Huntington’s disease	Case report	Improved severe depression, psychosis, and chorea after undergoing ECT
Garcia Ruiz (2021) [71]	To examine the efficacy of ECT in Huntington’s disease and PD	A brief review of extant evidence	Worse movement among patients with Huntington’s disease
Abeysundera et al. (2019) [73]	To establish the outcomes of administering ECT to a patient with Huntington’s disease	Patient case report	Worse movement among patients with Huntington’s disease
El-Sourady et al. (2022) [74]	To examine the efficacy of various therapies used to offer palliative care to individuals with Huntington’s disease	Scooping review	Improved patient’s depression when combined with venlafaxine
Adrissi et al. (2019) [72]	To review the use of ECT for Huntington’s disease patients with medication-refractory depression to psychosis to ascertain its efficacy	A single-center retrospective case series	Better outcomes in depression, suicidal intentions, and agitation
Mori et al. (2021) [32]	To examine the efficacy of ECT in treating depression and neuroleptic malignant syndrome among patients with PD	Case report	Improved depression and malignant syndrome symptoms
Takamiya et al. (2021) [33]	To establish the efficacy of ECT in managing non-motor symptoms, such as depression and psychosis, among PD patients	A critical review of previous studies	Enhanced PD symptoms
Murayama et al. (2021) [34]	To examine the potential of ECT in improving psychiatric and motor symptoms among PD patients	Clinical trial	Enhanced PD symptoms
Rodin et al. (2021) [35]	To examine the impact of ECT on PD-related psychiatric and motor symptoms, including motor response	Clinical data analysis	Enhanced PD symptoms
Subramanian et al. (2022) [75]	To examine the efficacy of ECT in managing drug-resistant depressive symptoms	Review of cases	Enhanced drug-resistant depressive symptoms
Goegan et al. (2022) [76]	To establish the efficacy of ECT in achieving long-term outcomes among individuals experiencing depressive symptoms and functional disability	Clinical trial	Enhanced depressive symptoms
Guillet et al. (2020) [57]	To establish the efficacy of ECT in improving neurological symptoms among dopa-responsive dystonia patients	Case report	Dopa-responsive dystonia
Repple et al. (2020) [77]	To investigate the impact of ECT on the white matter changes over time among major depressive disorder patients	Clinical trial	Improved major depressive symptoms
Baldinger-Melich et al. (2017) [4]	To appraise the role of ECT in a patient with Parkinsonian syndrome	Case report	Improved Parkinsonian syndrome symptoms
Calderon-Fajardo et al. (2015) [5]	To examine the role of ECT in PD	Clinical trial	ECT is effective in treating refractory neuropsychiatric symptoms
Erickson et al. (2015) [6]	To establish the efficacy of ECT in treating deep brain stimulator-related symptoms	Case report	ECT improves symptoms associated with deep brain stimulator
Bui et al. (2011) [7]	To describe an increase in dopamine intake after ECT	Clinical design	Improvement in PD symptoms
Usui et al. (2011) [8]	To examine the role of ECT in PD	Clinical trial	Improvement in PD symptoms
Fernandez-Corcuera et al. (2011) [9]	To establish the efficacy of ECT in PD and schizophrenia	Clinical trial	Enhancement in associated symptoms
Ueda et al. (2010) [10]	To establish the efficacy of ECT in improving PD symptoms	Clinical trial	Improved psychiatric symptoms
Nasr et al. (2011) [11]	To examine the use of ECT in treating PD with deep brain stimulation	Case report	Improved outcomes
Chiu (2009) [12]	To examine the role of ECT in managing PD and Capgras syndrome symptoms	Clinical trial	Positive outcomes
Kamigaichi et al. (2009) [13]	To examine the role of ECT in treating catatonia among PD patients	Care report	Improved catatonia symptoms
Bailine et al. (2008) [14]	To establish the role of ECT in managing depression in PD patients	Clinical trial	Suppressed depression
Balke et al. (2007) [15]	To examine the efficacy of ECT in treating symptoms associated with PD	Case presentation	Improved PD symptoms
Chou et al. (2005) [16]	To examine the efficacy of ECT in treating symptoms associated with PD	Case presentation	Improved PD symptoms
Shulman et al. (2003) [17]	To scrutinize the efficacy of ECT in treating symptoms associated with PD	Case presentation	Improved PD symptoms
Cunningham et al. (2016) [18]	To establish the efficacy of ECT in treating symptoms associated with PD	Case study	Improved PD symptoms
Gadit et al. (2012) [19]	To examine the efficacy of ECT in treating symptoms associated with PD	Case presentation	Improved PD symptoms
Nishioka et al. (2014) [20]	To examine the efficacy of ECT in treating symptoms associated with PD	Case presentation	Improved PD symptoms
Berg (2011) [21]	To examine the efficacy of ECT in treating symptoms associated with PD	Case presentation	Improved PD symptoms
Ducharme et al. (2011) [22]	To examine the efficacy of ECT in treating symptoms associated with PD	Case presentation	Improved PD symptoms
Meagher et al. (2006) [23]	To examine the efficacy of ECT in treating symptoms associated with PD	Case presentation	Improved PD symptoms
Ozer et al. (2005) [24]	To examine the efficacy of ECT in treating symptoms associated with the neuroleptic malignant syndrome	Case presentation	Improved neuroleptic malignant syndrome symptoms
Muralidharan et al. (2011) [25]	To examine the efficacy of ECT in treating symptoms associated with PD	Case presentation	Improved PD symptoms
Marino and Friedman (2013) [26]	To examine the efficacy of ECT in treating symptoms associated with PD	Case presentation	Improved PD symptoms
Pintor et al. (2012) [27]	To examine the efficacy of ECT in treating symptoms associated with PD	Pilot study	Improved PD symptoms
Suzuki et al. (2006) [28]	To examine the efficacy of ECT in treating symptoms associated with PD	Case presentation	Improved PD symptoms
Mortier et al. (2013) [30]	To examine the efficacy of ECT in treating symptoms associated with PD	Case report and review	Improved PD symptoms
Baez and Avery (2011) [37]	To examine the efficacy of ECT in treating symptoms associated with an intracranial metallic object	Case presentation	Improved symptoms associated with a metallic object
Dastgheib et al. (2009) [38]	To examine the efficacy of ECT in treating symptoms associated with PD	Case report	Improved PD symptoms
Sadananda et al. (2013) [39]	To examine the efficacy of ECT in treating symptoms associated with PD	Case presentation	Improved PD symptoms
Sienaert and Peuskens (2005) [40]	To examine the efficacy of ECT in treating symptoms associated with treatment-refractory schizophrenia	Case presentation	Improved PD symptoms
Peng et al. (2013) [41]	To examine the efficacy of ECT in treating symptoms associated with PD	Case report and literature review	Improved PD symptoms
Nobuhara et al. (2004) [42]	To examine the efficacy of ECT in treating symptoms associated with tardive dyskinesia	Case presentation	Improved PD symptoms
Yasui-Furukori et al. (2014) [43]	To examine the efficacy of ECT in treating symptoms associated with tardive dyskinesia	Retrospective design	Improved tardive dyskinesia symptoms
Sharma et al. (2007) [44]	To examine the efficacy of ECT in treating symptoms associated with ocular dystonia	Case presentation	Improved PD symptoms
Guo et al. (2016) [47]	To examine the efficacy of ECT in treating symptoms associated with Tourette’s syndrome	Case report	Improved ocular dystonia symptoms
Dehning et al. (2011) [48]	To examine the efficacy of ECT in treating symptoms associated with Tourette’s syndrome	Case presentation	Improved Tourette’s syndrome symptoms
Morais et al. (2007) [49]	To examine the efficacy of ECT in treating symptoms associated with Tourette’s syndrome	Case presentation	Improved Tourette’s syndrome symptoms
Karadenizli et al. (2005) [50]	To examine the efficacy of ECT in treating symptoms associated with Tourette’s syndrome	Case presentation	Improved Tourette’s syndrome symptoms
Trivedi et al. (2003) [51]	To examine the efficacy of ECT in treating symptoms associated with Tourette’s syndrome	Case presentation	Improved Tourette’s syndrome symptoms
Rajashree et al. (2014) [52]	To examine the efficacy of ECT in treating symptoms associated with Tourette’s syndrome	Case presentation	Improved Tourette’s syndrome symptoms
Dhossche et al. (2010) [53]	To examine the efficacy of ECT in treating symptoms associated with catatonia	Case presentation	Improved catatonia symptoms
Strassnig et al. (2004) [54]	To examine the efficacy of ECT in treating symptoms associated with PD	Case presentation	Improved PD symptoms
Nakano et al. (2013) [58]	To examine the efficacy of ECT in treating symptoms associated with Tourette’s syndrome	Case presentation	Improved atrophy and bipolar symptoms
Obiora et al. (2012) [59]	To examine the efficacy of ECT in treating symptoms associated with atrophy and bipolar disorder	Case presentation	Improved dyskinesia symptoms
Azuma et al. (2010) [60]	To examine the efficacy of ECT in treating symptoms associated with dyskinesia with depression	Case presentation	Improved dyskinesia depression symptoms
Chia et al. (2014) [61]	To examine the efficacy of ECT in treating symptoms associated with psychiatric manifestations of multiple system atrophy	Systematic review	Improved multiple system atrophy syndrome symptoms
Vazquez et al. (2009) [62]	To examine the efficacy of ECT in treating symptoms associated with McLeod syndrome	Case presentation	Improved McLeod syndrome symptoms
Kushner et al. (2007) [63]	To examine the efficacy of ECT in treating symptoms associated with essential tremor	Case presentation	Improved essential tremor symptoms
Shioda et al. (2006) [64]	To examine the efficacy of ECT in treating symptoms associated with multiple system atrophy	Case series	Improved multiple system atrophy dystonia symptoms
Sienaert et al. (2009) [65]	To examine the efficacy of ECT in treating symptoms associated with dopa-responsive dystonia	Case presentation	Improved dopa-responsive dystonia symptoms
Vila-Rodriguez et al. (2014) [66]	To examine the efficacy of ECT in treating symptoms associated with deep brain stimulators	Case report	Improved deep brain stimulator symptoms
Quinn et al. (2014) [67]	To examine the efficacy of ECT in treating catatonia symptoms	Case report	Improved catatonia symptoms
Petit et al. (2016) [68]	To examine the efficacy of ECT in treating symptoms associated with PD	Case report	Improved PD symptoms
Rutherford (2012) [69]	To examine the efficacy of ECT in treating symptoms associated with chorea and prominent delusions	Systematic review	Improved chorea and prominent delusions symptoms
Baldinger et al. (2014) [80]	To examine the efficacy of ECT in treating symptoms associated with chorea and prominent delusions	Systematic review	Improved chorea and prominent delusions symptoms
Yadid et al. (2000) [81]	To examine the efficacy of ECT in treating symptoms associated with the neurobiology of depression	Review of animal model	Improved psychotic depression symptoms
Popeo et al. (2009) [82]	To examine the efficacy of ECT in treating symptoms associated with PD	Literature review	Improved PD symptoms
Mateos et al. (2007) [83]	To examine the efficacy of ECT in treating symptoms associated with PD	Clinical trial	Improved PD symptoms
Rizos et al. (2010) [84]	To examine the efficacy of ECT in treating antipsychotic symptoms	Case report	Improved psychotic depression symptoms
Sanacora et al. (2003) [85]	To examine the efficacy of ECT in treating depressed patients	Clinical trial	Improved depression symptoms
Fusar-Poli et al. (2010) [86]	To examine the efficacy of ECT in treating signs of psychosis	Multimodal imaging design	Improved psychotic depression symptoms
McCormick et al. (2007) [87]	To examine the efficacy of ECT in treating symptoms associated with psychotic depression	Clinical trial	Improved psychotic depression symptoms
McNally et al. (2004) [88]	To examine the efficacy of ECT in treating symptoms associated with PD	Case review	Improved PD symptoms
Katz et al. (2017) [89]	To examine the efficacy of ECT in treating symptoms associated with Tourette’s syndrome	Case report	Improved Tourette’s syndrome symptoms
Peroski et al. (2019) [90]	To examine the efficacy of ECT in treating symptoms associated with implanted deep brain stimulators	Review	Improved symptoms associated with implanted deep brain stimulators
Mughal et al. (2011) [91]	To examine the efficacy of ECT in the disease process	Clinical trial	Improved disease treatment process
Aziz et al. (2005) [92]	To examine the efficacy of ECT in treating depression	Analytic design	Improved depression symptoms
Bonds et al. (1998) [93]	To examine the efficacy of ECT in treating bipolar disorder	Clinical trial	Improved bipolar disorder symptoms
Cai et al. (2023) [78]	To explore the effectiveness of ECT in enhancing suicidal ideation among major depression patients	Controlled trial	The ECT group reported improvements in suicidal ideation after treatment

Discussion

It has been well documented that ECT is a promising intervention in the treatment of mood as well as thought disorders. An increasing amount of literature suggests the efficacy for a range of movement disorders with and without comorbid mood or psychotic disorders. Antidepressants and antipsychotics are mild-to-moderately effective in treating a comorbid mood disorder in those with a primary movement disorder, which is a neurological condition affecting the ability to control movements resulting from abnormalities in the brain’s motor system [2]. The study’s results show that ECT should be considered a treatment for PD and Parkinsonian disorders as well as a plethora of comorbid diseases, such as depression, anxiety, and others. Specific recommendations regarding the length of treatment might be hard to formulate owing to the use of multiple strategies for conducting ECT sessions; at the same time, it seems justified to state that around 7-20 sessions might be sufficient for inducing a positive effect in reducing the symptoms of movement disorders and comorbid diseases. There are multiple cases in which ECT has been documented to show improvement in movement disorders. PD has been the most reported, with a growing body of positive results. Other movement disorders, including drug-induced Parkinsonism, catatonia, and tic-like disorders, also have increasing reports of positive results. Significant improvement was measured by standardized scales, clinical observation, or reduction in LEDD without compromising the motor function of patients.

The publications examined in this systematic review delineate ECT as an effective intervention for symptoms associated with various movement disorders. Several studies confirmed a positive relationship between this therapy and PD [31-36]. After the sixth and seventh weeks, ECT significantly improved psychiatric, motor, and major depressive symptoms among PD patients [31]. This effect depicts the therapy as a possible complementary intervention for treating and managing PD symptoms. Besides, ECT has been reported to reduce the symptoms of depression and malignant syndrome among PD patients [32]. Depression and malignant syndrome are critical symptoms of PD that cause adverse health implications. Physicians can recommend ECT to PD patients for managing non-motor symptoms such as depression and psychosis. Notably, ECT has been linked to long-lasting improved outcomes in motor and psychiatric symptoms and a reduction in the daily dose of anti-PD drugs [34]. These positive outcomes can augment the quality of life of PD patients. The potential to reduce anti-PD drug reliance can significantly boost a patient’s quality of life.

This systematic review also found a positive impact of ECT on Tourette’s syndrome. Several studies have highlighted the efficacy of the intervention in managing various symptoms of Tourette’s syndrome [47-56]. ECT has also been found to be an effective complement to clozapine in managing treatment-resistant schizophrenia [55]. Thus, ECT can support clozapine in treating PD patients with drug-resistant schizophrenia. From a broader perspective, this evidence affirms the role of ECT in augmenting pharmacological medication to manage drug-resistant schizophrenia. This relationship is in line with the outcome of a systematic review of previous medical reports, which indicated that ECT has a positive outcome in Tourette’s syndrome and related conditions [56]. Patients with drug-resistant symptoms may benefit from this therapy through improved symptoms and overall quality of life.

The outcome of this systematic review also indicates a positive relationship between ECT and tardive dyskinesia. An analysis of six studies revealed that the therapy improves psychotic, mood, and major depressive symptoms [40-46]. ECT also leads to a decrease of at least 8 points in the AIMS score [45], although some studies report a 10-point increase or even a more significant improvement [44,46]. The improvement in the AIMS score is a significant indicator of positive outcomes in a patient’s psychotic and mood symptoms. These shreds of evidence imply that ECT can enable patients to register improvements in tardive tremor symptoms. Thus, the literature highlights the role of ECT in managing tardive tremors among patients with major depressive disorder. Overall, available evidence depicts ECT as an alternative treatment for major depressive disorder in patients with tardive tremors.

The analyzed data also confirm a positive association between ECT and other movement disorders. It seems justified to assume based on the review’s findings that this intervention might improve symptoms associated with multisystem atrophy, Huntington's disease, paroxysmal non-kinesigenic dyskinesia, and cervical dystonia [47-79]. The analysis revealed that the intervention positively impacted the treatment of multisystem atrophy, including motor and psychiatric symptoms following ECT [48,50,53]. Additionally, the systematic review established a positive effect of ECT in dystonia treatment. Notably, the intervention is associated with enhanced outcomes in two critical symptoms, namely, delusional depression and agitation [54,55]. However, the review found no evidence to support the impact of ECT on motor symptoms among patients with dopa-responsive dystonia [54]. On the same token, the therapy was confirmed to be effective in leading to stable movement among patients with cervical dystonia.

For example, ECT can lead to improved neurological outcomes [57]. This evidence authenticates the role of ECT in treating neurological symptoms among patients with dopa-responsive dystonia. The reviewed studies also found a positive link between ECT psychiatric symptoms associated with Huntington’s disease [70-73]. Notably, the intervention reduces the symptoms of severe depression, psychosis, and chorea among patients with this disorder [70]. ECT was also linked with additional positive effects, including alleviation of depression, suicidal intentions, and agitation, resulting in successful hospital discharge [71]. The multiplicity of these positive outcomes validates the need to combine ECT with pharmacological interventions to treat patients with Huntington’s disease. However, some reviewed studies indicated that ECT worsens movement capabilities and performance [72,73]. This negative nexus underlines the need to exercise caution when integrating ECT into the grand plan for treating Huntington’s disease. Further research is required to comprehend the best approaches to ECT utilization without adversely affecting movement performance.

Available evidence does not show conclusive evidence for a positive impact of ECT on neuroacanthocytosis symptoms. Simultaneously, one of the studies reported that ECT improves the disorder’s psychiatric symptoms for some months before showing a progressive deterioration until the patient succumbs [62]. The review did not find recent data on the effectiveness of ECT among patients with Alzheimer’s disease post-intervention. Indeed, ECT does not have a statistically significant outcome on symptoms related to Alzheimer’s [79], especially in managing agitation and aggression. Therefore, further research is needed to understand how ECT can be improved to augment its usefulness in treating these symptoms.

While the exact mechanism of improvement has not been elicited, there have been multiple hypotheses regarding mood and motor improvement with ECT. Improvement may be secondary to serotonergic transmission and activation of mesocorticolimbic pathways [80]. Noradrenergic effects may also be relevant to the development of movement disorders and mood symptoms [52,80]. Some propose receptor alteration or disruption of the blood-brain barrier leading to increased levodopa intake as possible causes for the improvement in motor symptoms [3,81,82]. It has been noted that there is a decrease in dopamine active transporter (DAT) secondary to neurodegenerative disease [83]. If not secondary to neurogenesis, possible upregulation of DAT and increased density may contribute to increased dopamine responsiveness following ECT.

Increased striatal DAT density has also been associated with improvement in tardive dyskinesia [84]. Proton magnetic resonance spectroscopy has identified the upregulation of GABAergic neurotransmission post-ECT [85]. An increase in striatal GABA and prevention of hypersensitization of postsynaptic dopamine receptors may also play a role in improving dyskinesias [41]. Striatal GABA increase may also improve neurological symptoms of multiple system atrophy, such as bradykinesia, tremors, rigidity, dyskinesia, depression, and anxiety [63]. ECT has effects on various neurological systems related to neurotransmitter release, receptor binding, and overall neurotransmission. There have been studies suggesting upregulation of postsynaptic 5HT1A receptors in the hippocampus and 5HT2A in the prefrontal cortex, with an overall global reduction similar to antidepressant medication observations. Activation of the dopamine system by ECT is also suggested by current literature [80].

Alterations in regional cerebral blood flow have been documented through imaging and hypothesized as contributing to positive effects associated with ECT [60]. One study used single-photon emission computed tomography (SPECT) imaging and observed changes in regional cerebral blood flow following ECT. Based on the results, it is postulated that the increase in cerebral blood flow to the right middle frontal gyrus post-ECT was negatively correlated with psychotic symptoms measured by the scale for assessment of positive symptoms [8]. This is consistent with a multimodal imaging study with functional MRI and positron emission tomography (PET) that has noted the development of psychosis associated with dysfunction in the prefrontal, middle frontal, and striatum [86]. Imaging studies using PET revealed increased metabolism in cortical areas which significantly correlated with reductions of HDRS scores as well as reduction of positive symptoms [87]. An additional study using SPECT imaging to measure cerebral blood flow and metabolic changes noted bitemporal lead placement activated frontotemporal and parietal association cortex. Bifrontal leads activated the prefrontal cortex and right unilateral placement caused minimal changes in left frontotemporal regions. Midline subcortical networks were also activated with ECT. Post-treatment electroencephalogram showed increased slow wave delta in the frontal and temporal regions [88].

Electroconvulsive Therapy and Intracranial Objects

Movement disorders often become unresponsive to medication treatment, or individuals may experience intolerable side effects from medication, which might happen due to underlying medical conditions, disease progression, or the need to increase the dosage of medication over time. Deep brain stimulation (DBS) is a neurosurgical procedure in which a brand pacemaker is implanted in the brain to deliver electrical stimulation to specific areas of the brain that are responsible for movement function. Although it has been approved for multiple movement disorders such as PD, dystonia, or tremors, there is a surgical risk associated with placement. Mood, anxiety, and psychotic disorders associated with DBS placement, revision, or electrical events are not rare and can be refractory to pharmacological treatment [14,16,18,57]. Predisposition gives cause for close monitoring for mood, anxiety, or psychotic symptoms. Theoretical risks of damage to the device, activation, heating, or movement of electrodes have not been observed in any reports. ECT has been shown to be safely administrable in combination with a DBS device to successfully treat movement disorders and associated psychiatric symptoms, such as depression and anxiety [6,11,14,16,18,22,29,56,57,89]. Lead placement varied from bifrontal, bilateral [11,14,18,22,56], right unilateral [29,57], and left anterior right temporal [6]. DBS device was turned off or the voltage was set to zero in all but one report.

There was no apparent interference to a DBS device or device disruption of the effectiveness of ECT in all cases reviewed [6,11,14,16,18,22,29,56,57,89]. Imaging was used to monitor for shifting of the electrodes, and no movement was identified [16,29]. Given the risks associated with DBS placement, scientists might explore the use of ECT as a non-invasive alternative [48]. There was one documented case report of a patient with PD, acute mania, and an intracranial metallic fragment in which ECT was successfully used. The literature review concluded using ECT in patients with intracranial metal can be safe. Objects included DBS electrodes, fixation systems, bullet fragments, titanium plating, steel plating, metal prostheses, or aneurysmal coils and clips [90]. In this review, no evidence was found showing that ECT with an intracranial device was unsafe. However, the sample size was limited, some precautions were taken, there are no large-scale studies, and positive outcomes are more likely to be reported compared to negative ones.

Efficacy and Tolerability of Pharmacotherapy Versus Electroconvulsive Therapy

Movement disorders and subsequent pharmacological treatment can lead to psychosis or mood disorders; furthermore, severe psychotic disorders and treatment can lead to movement disorders. Both scenarios might occur as a result of the long-term use of certain medications, such as dopamine agonists or levodopa, the use of medications blocking dopamine receptors, and the underlying susceptibility of some patients with movement disorders to psychiatric symptoms. Medication working through metabolic changes and signaling systems can take time to make changes in the brain. Electrical stimulation can induce rapid changes in cortical and subcortical areas, making it an alternative to pharmacological treatments. In regards to safe administration, ECT has been shown to be effective with a low risk of complications, with a majority of reports showing positive outcomes in both movement and mood disorders independent of each other [18]. In addition to limited risk, ECT has also been found to be protective against olanzapine-induced Parkinsonism [38]. Other progressive neurodegenerative disorders and the role of ECT were studied with limited results.

Animal studies documented neuroprotective properties against huntingtin protein, which led to slowed progression and improved outcomes [91]. There is an unclear role of pharmacotherapy in multisystem atrophy; while ECT will not arrest the progression of many movement disorders, there are minimal side effects, no absolute contraindication, or risk of extrapyramidal symptoms. There are documented improvements in quality of life and cost-effectiveness. In addition to efficacy, cost-effectiveness has been demonstrated through multiple case reports, case series, and cost-utility analyses [92,93]. One study presented a case of bipolar disorder which documented a greater than 50% cost reduction associated with m-ECT [73]. ECT should be considered before the last line or for disorders refractory to medication management.

Limitations

The findings of this study are subject to a set of limitations. In particular, it is important to point out that the studies reviewed in the research lacked standardized protocols. As a result, it was hard to compare their findings with each other. The scientists reported multiple ECT administration techniques, leading to high variability in settings and outcomes. In particular, while some scholars followed consistent protocols and reported changes in standardized scores in a large sample, others focused on small case studies of single patients. Furthermore, the review also displays a high variability in the comorbid psychiatric conditions. These limitations are vital components of the study’s research context that must be taken into account when applying its results for further research.

## Conclusions

This literature review sought to evaluate the previously published scholarly literature to establish the efficacy of ECT in treating various movement disorders. Most reviewed studies reported positive associations between this intervention and many movement disorders. Available evidence indicates that ECT alleviates the symptoms associated with PD by enabling patients to manage depression, malignant syndrome symptoms, and other psychiatric clinical signs. Similarly, ECT has a positive effect on Tourette’s syndrome. Patients with drug-resistant symptoms are likely to experience reduced symptoms and an improvement in the overall quality of life post-intervention.

Additionally, this systematic review established that ECT can reduce tardive dyskinesia symptoms, including therapy that reduces the symptoms of psychotic, mood, and major depression. The reviewed data also provide a premise to assume that there might be a positive association between ECT and other movement disorders. Notably, the intervention may alleviate the severity of symptoms associated with multisystem atrophy, Huntington’s disease, paroxysmal non-kinesigenic dyskinesia, essential tremor, and cervical dystonia. In comparison with older reviews of the use of ECT in patients with movement disorders, this study offers more details about the specific conditions maximizing the effectiveness of ECT sessions, highlights the positive effects of ECT on reducing symptoms of specific comorbid diseases, and provides compelling evidence for the effectiveness of ECT in alleviating the symptoms of mental health disorders in PD patients and patients with other movement disorders. In addition, it should be noted that this study relies on recent literature; therefore, its findings are up-to-date.

However, this systematic review revealed that ECT does not have a long-lasting impact on neuroacanthocytosis symptoms. Instead, the intervention improves the disorder’s psychiatric symptoms in the short term before showing a progressive deterioration until the patient’s death. Additionally, ECT has a negative association with aggression and agitation, two of the most critical symptoms of Alzheimer’s disease. Overall, a large body of research reports a positive association between ECT and mood disorders and movement disorders. While further research is vital in eliminating the few gray areas, the insights from this systematic review depict ECT as a promising intervention and potential alternative to pharmacological medications. Based on the reviewed evidence, ECT is an effective and safe non-pharmacological alternative treatment approach for multiple movement disorders.
